# Association of stress hyperglycemia with clinical outcomes in patients with ST-elevation myocardial infarction undergoing percutaneous coronary intervention: a cohort study

**DOI:** 10.1186/s12933-023-01812-9

**Published:** 2023-04-12

**Authors:** Qu-Cheng Wei, Yu-wen Chen, Qi-Yue Gao, Kai-Da Ren, Ya-Bin Liu, Fan He, Jia-Tong Shi, Jun Jiang

**Affiliations:** Department of Cardiology, Second Affiliated Hospital, Zhejiang University School of Medicine, Hangzhou, China

**Keywords:** Stress hyperglycemia, ST elevation myocardial infarction, In-hospital death, All cause mortality

## Abstract

**Background:**

In recent years, several studies have demonstrated that stress hyperglycemia is significantly associated with poor prognosis in patients diagnosed with acute coronary syndrome (ACS). In the present study, we aimed to investigate the potential associations between various markers of stress hyperglycemia, such as admission blood glucose (ABG), fasting blood sugar (FBS), and stress hyperglycemia ratio (SHR) with different definitions, and the occurrence of adverse cardiovascular events in patients diagnosed with ST-elevation myocardial infarction (STEMI) who have undergone percutaneous coronary intervention (PCI).

**Methods:**

Our study enrolled a total of 1099 patients diagnosed with STEMI who underwent PCI from 2016 to 2021. The primary outcomes of this study were in-hospital death and all-cause mortality.

**Results:**

Stress hyperglycemia was associated with a higher incidence of in-hospital death (ABG OR: 1.27 95% CI 1.19–1.36; FBS OR: 1.25 95% CI 1.16–1.35; SHR1 OR: 1.61 95% CI 1.21–2.14; SHR2 OR: 1.57, 95%CI 1.22–2.01; SHR3 OR: 1.59, 95%CI 1.24–2.05) and all-cause mortality (ABG HR: 1.10, 95% CI 1.07–1.14; FBS HR: 1.12, 95 CI 1.07–1.17; SHR1 HR: 1.19 95% CI 1.03–1.39; SHR2 HR: 1.28, 95%CI 1.14–1.44; SHR3 HR: 1.29, 95%CI 1.14–1.45) after adjusting for ischemic time, age, gender, BMI, hypertension, hyperlipidemia, diabetes mellitus (DM), current smoking history, chronic kidney disease (CKD), previous history of coronary artery disease (CAD), atrial fibrillation (AF), heart failure (HF), stroke, cancer, culprit vessel, multi-vessel disease. These associations exhibited a non-linear, J-shaped pattern, wherein the risk significantly increased when the ABG and FBS levels exceeded 5mmol/L. Moreover, the inflection point for SHR was estimated to be 1.2.

**Conclusions:**

Stress hyperglycemia was significantly associated with an increased risk of in-hospital death and all-cause mortality in STEMI patients treated with PCI. Stress hyperglycemia should be considered a high-risk prognostic marker in all STEMI patients, regardless of with or without diabetes.

**Supplementary Information:**

The online version contains supplementary material available at 10.1186/s12933-023-01812-9.

## Introduction

Coronary artery disease (CAD) is a significant global health concern and the primary cause of mortality worldwide [1]. While the increased utilization of percutaneous coronary intervention (PCI) and the widespread standardization of drug therapy have contributed to a substantial reduction in the mortality rate of ST-elevation myocardial infarction (STEMI) [2, 3], the incidence of both in-hospital and long-term mortality remains alarmingly high, ranging from 4–12% [1].

Stress hyperglycemia, characterized by a transient increase in blood glucose levels in response to illness-related stress, is a common occurrence among critically ill patients [4]. According to the Clinical Guidelines of the American Heart Association and the Endocrine Society, stress hyperglycemia is defined as a blood glucose level ≥ 140 mg/dl (7.8mmol/L) in both diabetic and non-diabetic patients [5]. Previous research has demonstrated a correlation between blood glucose levels and short- and long-term outcomes in cases of acute coronary syndrome (ACS) [6–10]. However, admission blood glucose (ABG) or fasting blood sugar (FBS) measurements may not provide a complete representation of the acute hyperglycemic state, which can be influenced by chronic glucose status. As a result, several investigations have proposed the stress hyperglycemia ratio (SHR) as an indicator of acute hyperglycemic state and a predictor of poor prognosis in ACS patients [11–13] .

Despite growing interest in stress hyperglycemia as a potential prognostic factor in STEMI patients, the available data remain limited. Consequently, the present study was designed to investigate the association between different indicators of stress hyperglycemia and short-term and long-term adverse outcomes in STEMI patients undergoing PCI.

## Methods

### Study design and population

This study was a single-center retrospective, observational cohort study. This study was performed according to the Declaration of Helsinki and World Health Organization guidelines. The study protocol was approved by the local ethics committee.

Inclusion criteria for this study comprised two factors: patients aged 18 years or older who had suffered STEMI and had undergone PCI. Conversely, exclusion criteria for this study were as follows: patients with missing critical laboratory data, specifically glucose or glycated hemoglobin measurements, and patients with a life expectancy of less than 1 year. The diagnosis of STEMI was based on two criteria: (1) the presence of typical clinical symptoms of myocardial ischemia and (2) new ischemic changes observed in electrocardiograms, specifically new ST-segment elevation in two contiguous leads (V2-V3 leads: ≥2 mm in men ≥ 40 years, ≥ 2.5 mm in men < 40 years, ≥ 1.5 mm in women regardless of age; other leads: ≥ 1 mm).

Cardiac intervention was conducted using either a radial or femoral approach, as determined by the treating physician. Patients received a loading dose of 300 mg of aspirin and either 300 mg of clopidogrel or 180 mg of ticagrelor. Heparin was administered as an initial bolus of 70-100IU/kg body weight, with an additional dose of 1000IU administered hourly thereafter. The decision to administer glycoprotein IIb/IIIa antagonists was left to the discretion of the operator.

### Data collection and definitions

We collected comprehensive baseline demographic and clinical data for all patients enrolled in this study. The demographic data we gathered included ischemic time, age, sex, body mass index (BMI), hypertension, diabetes mellitus (DM), hyperlipidemia, chronic kidney disease (CKD), smoking status, and previous history of CAD, atrial fibrillation (AF), heart failure (HF), stroke, cancer. The diagnosis of diabetes was established based on several criteria, including previous medical diagnosis, use of oral hypoglycemic drugs or insulin, or a hemoglobin A1c (HbA1c) level exceeding 6.5% [14]. Clinical data we recorded included laboratory and angiographic characteristics, as well as drug regimens administered both during hospitalization and at discharge. Multi-vessel disease was defined as luminal stenosis of at least 70% in at least two major epicardial coronary arteries.

The SHR was calculated using the following equations: (1) SHR1, which was obtained by dividing ABG by the estimated average glucose level. (2) SHR2, which was obtained by dividing FBS by the HbA1c level. (3) SHR3, which was obtained by dividing FBS by the estimated average glucose level. The estimated average glucose level was calculated using the following formula: estimated average glucose (mmol/L) = 1.59 × HbA1c (%) − 2.59.

### Follow-up and outcome definitions

The primary outcomes of this study were in-hospital death and all-cause mortality following STEMI. The secondary outcome was comprised of major adverse cardiac and cerebrovascular events (MACCE), which included all-cause mortality, unplanned revascularization, and stroke. Unplanned revascularization was defined as either ischemia-driven or clinic-driven revascularization of coronary arteries. Stroke was defined as either hemorrhagic or ischemic stroke or transient ischemic attack.

Follow-up data on clinical outcomes were obtained by trained independent investigators through telephone contacts, outpatient visits, or a thorough review of medical records. The clinical outcomes were adjudicated by well-trained independent physicians who were blinded to the study.

### Statistical analyses

Continuous variables were presented as means ± standard deviation (SD) for normal distribution and medians (interquartile range) for abnormal distribution. For comparison between different groups, either Student’s t-test or the Mann-Whitney U test was used, as appropriate. Categorical variables were presented as quantities and percentages. For comparison between different groups, either the chi-square test or Fisher’s exact test was used, as appropriate. The association between different stress hyperglycemic indicators and in-hospital death was confirmed by univariable and multivariable logistic regression model, whereas the effect of various stress hyperglycemic indicators on all-cause mortality was verified with Cox proportional regression model. We adjusted for potential confounders, such as ischemic time, age, gender, BMI, hypertension, hyperlipidemia, diabetes, current smoking history, CKD, previous history of CAD, AF, HF, stroke, cancer, culprit vessel, and multi-vessel disease with each model. The Kaplan-Meier method was used to estimate the event-free survival rates in different groups with log-rank test for further comparison. We employed the generalized additive model (GAM) to evaluate the potential non-linear association under investigation. The Log-likelihood ratio test was utilized to detect the inflection point that links the segments. Specifically, the one-line (non-segmented) model was compared with the maximum likelihood, and a two-step recursive method was implemented to achieve this objective. Subgroup analyses were undertaken to assess the effect of stress hyperglycemic indicators on in-hospital death and all-cause mortality for specific patients with comorbidities, particularly diabetes mellitus. A two-sided p-value < 0.05 was considered statistically significant for all analyses. All data in this study were processed using the statistical software packages R (http://www.R-project.org, The R Foundation) and Empower Stats (http://www.empowerstats.com, X&Y Solutions, Inc., Boston, MA).

## Results

### Baseline characteristics

Between June 2016 and June 2021, a total of 1099 STEMI patients were included in this study. The mean age of the participants was 62.55 ± 13.58 years, with 81.47% of patients being men. The average body mass index was 24.35 ± 8.70 kg/m^2^, and 59.76% of patients had hypertension. Additionally, 27.07% of patients were diagnosed with diabetes, and 34.70% of patients had hyperlipidemia. More than half of the participants were current smokers. The participants were categorized into four groups based on arterial blood glucose (ABG): quartile 1 (n = 274), ABG ≤ 6.78mmol/L; quartile 2 (n = 274), 6.78mmol/L < ABG ≤ 8.02mmol/L; quartile 3 (n = 276), 8.02mmol/L < ABG ≤ 10.81mmol/L; and quartile 4 (n = 275), ABG > 10.81mmol/L. Detailed baseline characteristics of the study population are presented in Table 1.

**Table 1:** Baseline demographic and clinical data

	ABG				
	Q1 (n = 274)	Q2 (n = 274)	Q3 (n = 276)	Q4 (n = 275)	*p*
Ischemia time, hours	8.00 (4.24, 24.00)	6.17 (3.25, 14.24)	5.93 (3.33, 10.88)	6.00 (3.64, 16.05)	0.004
Age, years	60.31 ± 14.92	61.86 ± 13.14	63.58 ± 12.89	64.39 ± 12.96	0.004
Gender, male, n(%)	235 (85.45)	234 (85.40)	220 (79.71)	208 (75.64)	0.006
BMI, kg/m^2^	23.69 ± 3.42	24.27 ± 3.25	24.05 ± 3.14	24.44 ± 3.37	0.026
Smoking history, n(%)					0.023
Current	158 (57.45)	139 (50.73)	121 (43.84)	122 (44.53)	
Past	26 (9.45)	38 (13.87)	44 (15.94)	41 (14.96)	
Medical history, n(%)					
Hypertension	134 (48.73)	155 (56.57)	175 (63.41)	193 (70.18)	< 0.001
Diabetes	15 (5.45)	19 (6.93)	73 (26.45)	190 (69.09)	< 0.001
Hyperlipidemia	82 (29.82)	97 (35.40)	104 (37.68)	99 (36.00)	0.237
Previous CAD	14 (5.09)	12 (4.38)	16 (5.80)	27 (9.82)	0.040
Previous AF	14 (5.09)	13 (4.74)	14 (5.07)	15 (5.45)	0.986
Previous stroke	19 (6.91)	21 (7.66)	21 (7.61)	27 (9.82)	0.619
CKD	9 (3.27)	13 (4.74)	15 (5.43)	24 (8.73)	0.039
Previous HF	1 (0.36)	1 (0.36)	2 (0.72)	3 (1.09)	0.665
Previous cancer	8 (2.91)	12 (4.38)	15 (5.43)	15 (5.45)	0.433
Heart rate, bpm	80.44 ± 15.34	81.35 ± 14.12	83.39 ± 17.34	89.93 ± 19.64	< 0.001
SBP, mmHg	124.40 ± 19.90	123.64 ± 19.79	122.41 ± 19.49	123.46 ± 23.69	0.830
DBP, mmHg	77.36 ± 14.02	76.07 ± 14.39	77.70 ± 28.04	74.23 ± 15.32	0.208
SpO_2_, %	98.33 ± 1.73	98.12 ± 2.15	98.24 ± 4.44	96.97 ± 4.37	< 0.001
Laboratory examinations					
White blood cell, 10^9/L	10.13 ± 3.82	10.66 ± 3.37	11.20 ± 3.57	12.40 ± 4.80	< 0.01
Hemoglobin, g/L	142.34 ± 20.18	145.79 ± 20.87	142.46 ± 20.35	140.34 ± 21.08	0.007
Platelets, 10^9/L	211.58 ± 66.33	214.90 ± 67.93	221.22 ± 59.61	217.07 ± 70.85	0.198
Creatinine, µmol/L	73.00 (64.00, 89.00)	75.00 (64.00, 87.00)	77.00 (65.00,92.50)	83.00 (64.25, 114.00)	< 0.001
ABG, mmol/L	6.02 ± 0.56	7.36 ± 0.35	9.17 ± 0.74	15.72 ± 4.34	< 0.001
FBS, mmol/L	5.28 ± 0.89	5.84 ± 1.20	7.02 ± 2.40	10.53 ± 4.11	< 0.001
SHR1	0.91 ± 0.13	1.08 ± 0.13	1.22 ± 0.21	1.49 ± 0.40	< 0.001
SHR2	0.79 ± 0.13	0.85 ± 0.16	0.91 ± 0.27	1.02 ± 0.40	< 0.001
SHR3	0.91 ± 0.14	0.98 ± 0.18	1.07 ± 0.32	1.28 ± 0.48	< 0.001
HbA1c, %	5.83 ± 0.47	5.96 ± 0.52	6.50 ± 1.02	8.41 ± 1.95	< 0.001
Triglycerides, µmol/L	1.30 (0.95, 1.90)	1.38 (0.95, 1.98)	1.34 (1.00, 1.86)	1.48 (1.03, 2.09)	0.139
HDL, µmol/L	1.04 (0.91, 1.18)	1.02 (0.89, 1.20)	1.05 (0.92, 1.21)	1.00 (0.87, 1.18)	0.265
LDL, µmol/L	2.38 (1.92, 2.98)	2.56 (2.09, 3.12)	2.62 (1.95, 3.20)	2.43 (1.81, 3.06)	0.632
LVEF, %	55.73 ± 10.69	55.47 ± 10.85	54.21 ± 11.00	50.95 ± 12.94	< 0.001
Procedural information					
Culprit vessel, n(%)					0.002
LM	1 (0.36)	0 (0)	9 (3.26)	12 (4.36)	
LAD	152 (55.27)	153 (55.84)	126 (45.65)	135 (49.09)	
LCX	39 (14.18)	31 (11.31)	36 (13.04)	38 (13.82)	
RCA	83 (30.18)	90 (32.85)	105 (38.04)	90 (32.73)	
Multivessel disease, n(%)					0.003
Two-vessel	76 (27.64)	77 (28.10)	93 (33.70)	85 (30.91)	
Three-vessel	45 (16.36)	49 (17.88)	59 (21.38)	75 (27.27)	
TIMI flow, n(%)					0.020
0	169 (61.45)	188 (68.61)	209 (75.72)	187 (68.00)	
1	12 (4.36)	6 (2.19)	13 (4.71)	10 (3.64)	
2	27 (9.82)	19 (6.93)	10 (3.62)	19 (6.91)	
3	67 (24.36)	61 (22.26)	44 (15.94)	59 (21.45)	
Thrombectomy, n(%)	144 (52.36)	164 (59.85)	162 (58.70)	167 (60.73)	0.182
Number of stents	1.22 ± 0.46	1.21 ± 0.44	1.25 ± 0.51	1.23 ± 0.52	0.918
Diameter of stents	3.00 ± 0.46	3.05 ± 0.48	3.09 ± 0.48	2.98 ± 0.46	0.026
Length of stents	30 (24, 38)	30 (24, 38)	30 (24, 38)	30 (23, 38)	0.971
Medication, n(%)					
Statins	268 (98.53)	268 (98.89)	256 (97.71)	242 (96.80)	0.326
ACEI or ARB	232 (85.29)	229 (84.50)	219 (83.59)	197 (78.80)	0.199
β-Blocker	244 (89.71)	232 (85.61)	225 (85.88)	207 (82.80)	0.152
Anticoagulants	19 (6.99)	17 (6.27)	8 (3.05)	14 (5.60)	0.213

Data are means ± SD, median (interquartile range), or n (%)

Abbreviations: BMI: Body mass index; SBP: Systolic blood pressure; DBP: Diastolic blood pressure; SpO_2_: Oxygen saturation; FBS: Fasting blood sugar; SHR: Stress hyperglycemia ratio; HbA1c: Glycosylated hemoglobin, Type A1c; LDL-C: Low density lipoprotein cholesterol; HDL-C: High density lipoprotein cholesterol; RMWA: Regional wall motion abnormality; LVIDd: Left ventricular internal diameter at end-diastole; LVEF: Left ventricular ejection fraction; LM: Left main artery; LAD: Left anterior descending artery; LCX: Left circumflex artery; RCA: Right coronary artery; TIMI: Thrombolysis in myocardial infarction; ACEI: Angiotensin converting enzyme inhibitors; ARB: Angiotensin II receptor blockers

This study has revealed statistically significant differences between the four groups in several variables, including ischemic time, age, sex, BMI, smoking history, and diagnosis upon admission. Upon further analysis, the lowest body mass index was observed in quartile 1 compared to the other groups. Furthermore, quartile 4 demonstrated significantly higher levels of glycated hemoglobin and a greater proportion of patients with diabetes compared to the other groups.

### Clinical outcomes for adverse cardiovascular events

A standard follow-up visit was conducted for each patient in this cohort. The patients were enrolled consecutively, resulting in varying lengths of follow-up time, with a minimum of 9 months. The median follow-up time was 32.73 months (interquartile range 15.53, 49.90). Of the 1099 patients, 69 patients died during hospitalization. Moreover, there were 149 cases of all-cause death and 336 cases of MACCEs during follow-up, including 181 cases of unplanned revascularization and 19 cases of stroke.

**Fig. 1:**
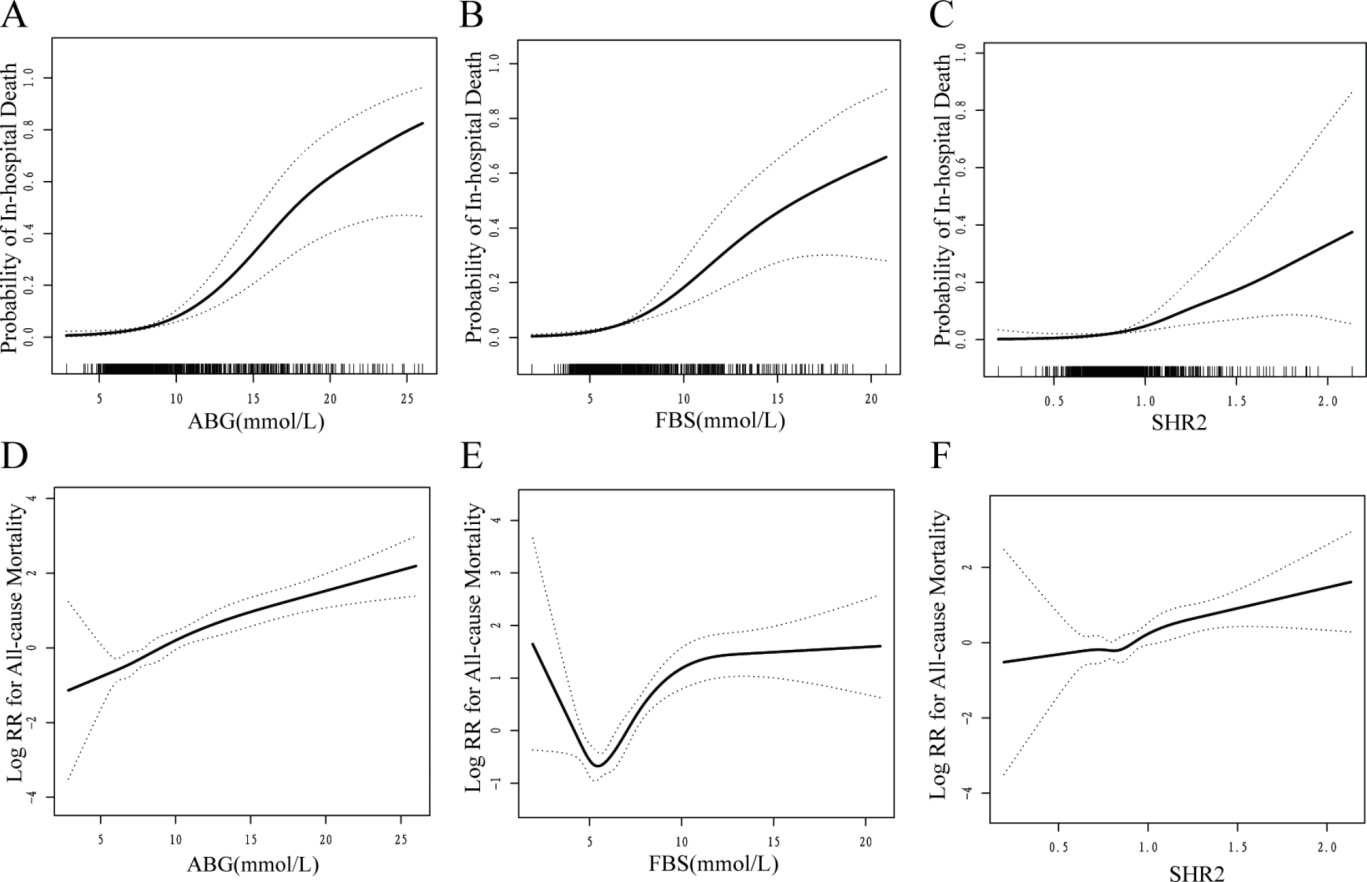
Association of stress hyperglycemia and adverse outcomes A: ABG and in-hospital death. B: FBS and in-hospital death. C: SHR2 and in-hospital death D: ABG and all-cause mortality. E: FBS and all-cause mortality. F: SHR2 and all-cause mortality. All analyses were adjusted for confounding factors including ischemic time, age, sex, BMI, hypertension, diabetes, hyperlipidemia, smoking status, previous CVD, previous AF, previous stroke, CKD, previous HF, cancer, culprit vessel, multi-vessel disease.

The application of logistic regression analyses revealed a significant association between hyperglycemia status, including ABG, FBS, and stress hyperglycemia ratios (SHR1, SHR2, SHR3), and a heightened risk of in-hospital mortality (ABG OR: 1.27, 95% CI: 1.19–1.36; FBS OR: 1.25, 95% CI: 1.16–1.35; SHR1 OR: 1.61, 95% CI: 1.21–2.14; SHR2 OR: 1.57, 95% CI: 1.22–2.01; SHR3 OR: 1.59, 95% CI: 1.24–2.05) (Table 2). Furthermore, these variables were evaluated as categorical variables, indicating that the rates of in-hospital death in quartile 4 were notably higher compared to quartile 1 (ABG Q4 OR: 23.33, 95% CI: 7.34–74.15; FBS Q4 OR: 10.10, 95% CI: 3.89–26.25; SHR1 Q4 OR: 20.77, 95% CI: 2.53-170.73; SHR2 Q4 OR: 9.95, 95% CI: 2.16–45.74; SHR3 Q4 OR: 19.65, 95% CI: 2.46-156.66), with a p value for trend of < 0.05. Refer to Supplementary Table 1 for further details. As ABG, FBS, and SHR were continuous variables, the examination of non-linear relationships was necessary. GAM were implemented to visually assess the relationships between hyperglycemia markers and in-hospital mortality risk. J-shaped associations were detected between FBS, SHR2, and in-hospital death, with an inflection point of 4.9mmol/L and 1.2, respectively (Fig. 1 and Supplementary Fig. 1).

**Table 2:** Multivariable Cox regression and Logistic regression analyses for different end points

	In-hospital death		All-cause mortality		Unplanned revascularization		MACCE	
	OR (95% CI)	*p*	HR (95% CI)	*p*	HR (95% CI)	*p*	HR (95% CI)	*p*
ABG	1.27 (1.19, 1.36)	< 0.001	1.10 (1.07, 1.14)	< 0.001	1.03 (0.99, 1.08)	0.130	1.08 (1.05, 1.11)	< 0.001
FBS	1.25 (1.16, 1.35)	< 0.001	1.12 (1.07, 1.17)	< 0.001	1.02 (0.96, 1.08)	0.480	1.07 (1.03, 1.11)	< 0.001
SHR1 (per SD)	1.61 (1.21, 2.14)	0.001	1.19 (1.03, 1.39)	0.019	1.08 (0.93, 1.25)	0.328	1.11 (1.00, 1.24)	0.051
SHR2 (per SD)	1.57 (1.22, 2.01)	< 0.001	1.28 (1.14, 1.44)	< 0.001	1.04 (0.88, 1.24)	0.610	1.13 (1.02, 1.24)	0.015
SHR3 (per SD)	1.59 (1.24, 2.05)	< 0.001	1.29 (1.14, 1.45)	< 0.001	1.05 (0.89, 1.25)	0.548	1.13 (1.03, 1.25)	0.012

Adjust for ischemia time, age, sex, BMI, hypertension, diabetes, hyperlipidemia, smoking status, previous CVD, previous AF, previous stroke, CKD, previous HF, cancer, culprit vessel, multi-vessel disease

Abbreviations: ABG: Admission blood glucose; FBS: Fasting blood sugar; SHR: Stress hyperglycemia ratio; MACCE: Major adverse cardiac and cerebrovascular events

**Fig. 2:**
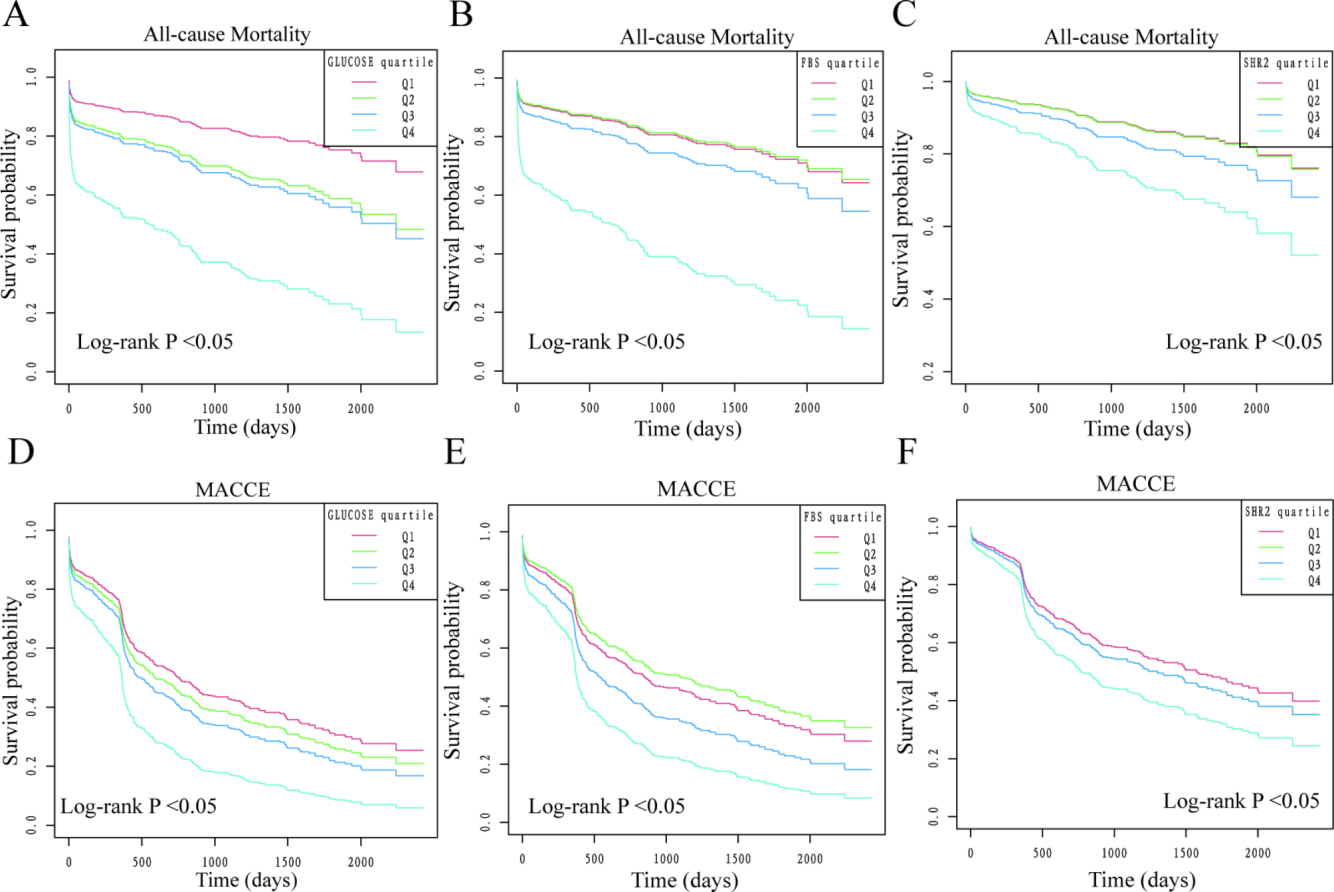
K-M analyses for all-cause mortality and MACCE. A: ABG and all-cause mortality. B: FBS and all-cause mortality. C: SHR2 and all-cause mortality. D: ABG and MACCE. E: FBS and MACCE. F: SHR2 and MACCE. MACCE: Major adverse cardiac and cerebrovascular events

The Cox regression analyses presented in Table 2 indicate that ABG, FBS, and SHR1,2,3, are potentially correlated with an increased risk of MACCEs. Specifically, the hazard ratios (HR) for ABG, FBS, SHR1,2,3, were found to be 1.08 (95% CI: 1.05–1.11), 1.07 (95% CI: 1.03–1.11), 1.11 (95% CI: 1.00-1.24), 1.13 (95% CI: 1.02–1.24), and 1.13 (95% CI: 1.03–1.25), respectively. Notably, this association was driven primarily by an increased risk of all-cause mortality, as evidenced by the HRs for ABG, FBS, SHR1,2,3, of 1.10 (95% CI: 1.07–1.14), 1.12 (95% CI: 1.07–1.17), 1.19 (95% CI: 1.03–1.39), 1.28 (95% CI: 1.14–1.44), and 1.29 (95% CI: 1.14–1.45), respectively. We conducted further assessments of various hyperglycemia markers as categorical variables. Kaplan-Meier analyses were used to generate cumulative survival rate curves for all-cause mortality and MACCE across four quantiles, with the highest HR observed in quantile 4 (all p < 0.05, except for SHR1, Fig. 2 and Supplement Fig. 2). Our results indicated that, when compared to individuals in quantile 1, those in quantile 4 had significantly higher multivariable-adjusted HR for all-cause mortality (ABG Q4 HR: 5.18, 95%CI: 2.83–9.50; FBS Q4 HR: 4.34, 95%CI: 2.45–7.71; SHR1 Q4 HR: 2.05, 95%CI: 1.13–3.70; SHR2 Q4 HR: 2.39, 95%CI: 1.37–4.15; SHR3 Q4 HR: 2.55, 95%CI: 1.42–4.58), with p values for trend being < 0.05. Supplement Table 1 provides detailed data on this topic. Additionally, our findings from GAM suggest a J-shaped association between FBS, SHR2 and all-cause mortality, with inflection points of 5mmol/L and 1.2 (Fig. 1 and Supplement Fig. 1).

### Sensitivity and subgroup analysis.

In this study, we utilized the definition of stress hyperglycemia, regardless of diabetic status, to divide patients into hyperglycemia and non-hyperglycemia groups, with a blood glucose cut-off of 140 mg/dl (7.8mmol/L). Regression analyses indicated that our results were consistent, with the multivariable-adjusted model showing that the odds ratios of in-hospital death for the stress hyperglycemic group versus the non-hyperglycemic group were 6.23 (95%CI: 2.79–13.89) for ABG and 12.27 (95%CI: 6.01–25.03) for FBS. Moreover, patients in the ABG or FBS hyperglycemic group had a higher risk of all-cause mortality compared to those in the ABG or FBS non-hyperglycemic group (for ABG, HR = 1.90, 95%CI: 1.28–2.82; for FBS, HR = 3.81, 95%CI: 2.50–5.79) (Supplementary Table 2). Supplement Fig. 3 provides detailed information on the Kaplan-Meier curves for all-cause mortality and MACCE among these groups.

**Fig. 3:**
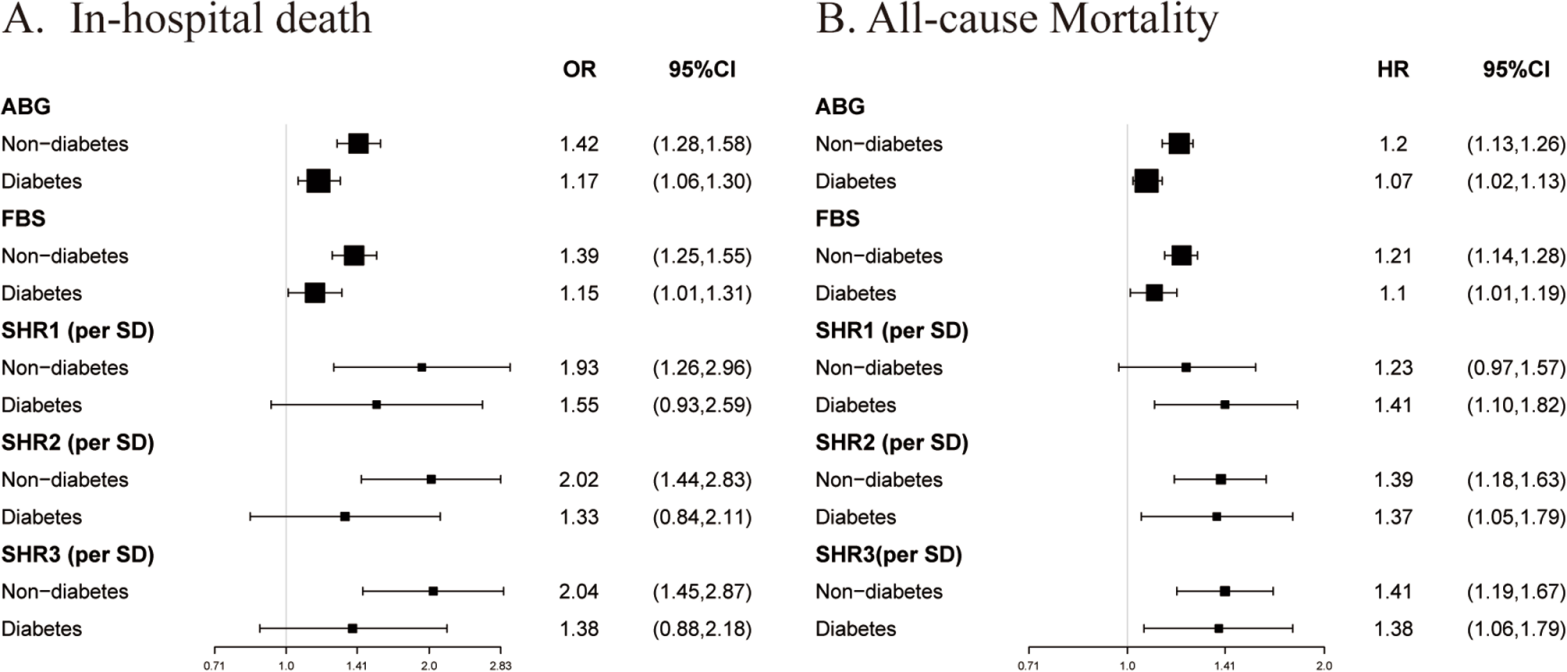
Subgroup analyses of stress hyperglycemia in diabetic and non-diabetic population A: In-hospital death. B: All-cause mortality. All analyses were adjusted for confounding factors including ischemic time, age, sex, BMI, hypertension, hyperlipidemia, smoking status, previous CVD, previous AF, previous stroke, CKD, previous HF, cancer, culprit vessel, multi-vessel disease

We conducted subgroup analyses to assess the association between ABG, FBS, and SHR and in-hospital death and all-cause mortality in different populations based on age and comorbidities such as DM, overweight, and hyperlipidemia (Fig. 3, Supplementary Figs. 4–6). Our findings suggest that the association between ABG, FBS, SHR1,2,3 and in-hospital death, as well as all-cause mortality, remained robust across both the diabetic and non-diabetic populations (Fig. 3).

## Discussion

The objective of this study was to investigate the potential correlation between stress hyperglycemia and prognosis in patients with STEMI who had undergone PCI. To achieve this goal, we conducted sensitivity analyses and trend tests to evaluate the relationship between commonly used stress hyperglycemia indicators, such as ABG, FBS, and SHR, and in-hospital as well as all-cause mortality. Our results demonstrated a consistent positive association between stress hyperglycemia and poor prognosis. Furthermore, subgroup analyses revealed that this relationship remained robust and consistent regardless of patients’ underlying conditions, including diabetes or overweight. Moreover, we found that the relationship between stress hyperglycemia and death during hospitalization or all-cause mortality was nonlinear and J-shaped, with an inflection point occurring at 5 mmol/L for ABG and FBS and at 1.2 for SHR. These findings suggest that stress hyperglycemia is an important predictor of poor outcomes in STEMI patients treated with PCI, and underscore the need for careful monitoring and management of glycemic control in this patient population.

### ABG, FBS and acute MI

Stress hyperglycemia triggers the production of inflammatory factors such as IL-6, IL-8, and TNF-alpha in the body, which can exacerbate atherosclerosis through various intracellular pathways [15, 16]. Furthermore, stress hyperglycemia increases thrombogenic activity, resulting in a hypercoagulable state [17–19]. Prior studies have established a positive relationship between stress hyperglycemia and death during hospitalization in critically ill patients [20–22]. And suggest that glycemic control may improve patient outcomes [23]. In acute coronary syndrome, Pasquale et al. followed 2704 patients with acute myocardial infarction for a mean period of 26 months, finding a significant positive association between ABG and in-hospital as well as all-cause mortality, regardless of diabetes mellitus status [6]. Nikolaos et al. conducted a study involving 309 consecutive patients with STEMI, with the primary endpoint being adverse cardiovascular and cerebrovascular events over a median follow-up period of 1.7 years [8]. The study found that ABG was independently associated with an increased risk of major cardiovascular events. The present study focuses on patients with STEMI treated with PCI, and our findings are consistent with previous research. After adjusting for various confounding factors, we observed a stable and significant positive association between poor prognosis and ABG or FBS levels, regardless of diabetes or overweight status. Additionally, we conducted a further analysis and found that the relationship between stress hyperglycemia and mortality was not linear but rather a J-shaped curve, with a threshold effect occurring at ABG and FBS levels greater than 5 mmol/L.

### SHR and acute MI

In recent years, certain scholars have raised concerns that ABG or FBS may be inadequate for fully and accurately assessing the acute phase state of stress hyperglycemia. To address this issue, Wei Xu et al. conducted a study involving 7476 consecutive patients with STEMI from 2001 to 2004 [12]. After 30 days of follow-up, SHR was independently associated to the risks of major adverse cardiovascular events and all-cause mortality. However, in this study, more than 50% patients received thrombolytic therapy. Jie Yang et al. consecutively enrolled 5562 ACS patients who underwent drug-eluting stent implantation with a median follow-up of 28.3 months [11]. The results demonstrated that there were a J-shaped association between SHR and early and late cardiovascular outcomes. However, in their study, STEMI patients were less than 10%. Moreover, there were several equations to calculate SHR [24]. The present study builds upon previous research and offers further insights into the topic. Notably, there exists a lack of consensus regarding the definition of SHR in the literature. To address this issue, we conducted a sensitivity analysis and subgroup analysis on the three most commonly employed definitions of SHR, and the results demonstrate stability and consistency after controlling for confounding variables. Additionally, we employed a GAM approach to examine the relationship between SHR and hospitalization-related mortality as well as all-cause mortality. Our findings reveal a nonlinear J-shaped relationship between SHR and prognosis. Through threshold effect analysis, we identified a significant increase in the risk of mortality during hospitalization and all-cause mortality when SHR2 exceeded 1.2. Overall, our study contributes to the existing body of knowledge on SHR and its relationship with mortality outcomes.

To the best of our knowledge, this study is the first to investigate the relationship between different stress hyperglycemia criteria and in-hospital as well as all-cause mortality in patients with STEMI treated with PCI. In contrast to prior research, we consecutively enrolled patients who received PCI rather than thrombolytic therapy. The vast majority of these patients received a 2nd generation drug-coated stent and an antiplatelet regimen consisting of aspirin plus ticagrelor, rather than the previous 1st generation drug-coated stent and aspirin plus clopidogrel antiplatelet therapy. Patients with heavy thrombus loads also received tirofiban antiplatelet therapy. Additionally, this study conducted extensive sensitivity analyses, including analyses of various definitions of stress hyperglycemia and SHR, evaluations of variables as both continuous and categorical variables, trend tests, and subgroup analyses for the presence or absence of diabetes mellitus and overweight. Our findings revealed a stable and consistent positive correlation between stress hyperglycemia and poor prognosis in STEMI patients. Furthermore, we conducted further analysis and found that the relationship between stress hyperglycemia and mortality was nonlinear and J-shaped. When ABG, FBS, and SHR exceeded the threshold inflection point, the risk of both in-hospital death and all-cause mortality was significantly higher. These results emphasize the importance of careful glycemic control in STEMI patients treated with PCI and suggest that maintaining stress hyperglycemia levels around the identified threshold values may improve patient outcomes.

### Limitations

Our results should be interpreted cautiously due to some limitations. The present study was carried out in a single center with a relatively small sample size, and the existing selection bias and other potential confounders may affect the results. Second, there was a lack of data on the use of hypoglycemic therapy in the follow-up period; thus, we could not evaluate their impact on the outcomes of STEMI patients. Furthermore, it is plausible that pre-diabetic patients exhibit distinct blood glucose variability compared to non-diabetic patients. Nonetheless, the failure to distinguish pre-diabetic patients from non-diabetic patients in this study precluded the analysis of pre-diabetic STEMI patients as a distinct subgroup, thereby hindering the identification of a potentially unique patient population.

## Conclusion

Our results indicate a significant positive correlation between stress hyperglycemia and increased risk of in-hospital death and all-cause mortality, regardless of with or without DM. Notably, we observed a non-linear J-shaped relationship between stress hyperglycemia and mortality outcomes. These findings highlight the potential clinical significance of stress hyperglycemia in predicting adverse outcomes in STEMI patients. However, further research is warranted to determine the diagnostic threshold for stress hyperglycemia and to explore its predictive value on an expanded scale. Therefore, we recommend a large-scale, multi-center, prospective cohort study to provide more robust evidence regarding the role of stress hyperglycemia in STEMI patients’ outcomes.

## Electronic supplementary material

Below is the link to the electronic supplementary material.

Supplementary Material 1

Supplementary Material 2

Supplementary Material 3
Supplement Figure 1. Association of stress hyperglycemia and adverse outcomes.
A: SHR1 and in-hospital death. B: SHR3 and in-hospital death. C: SHR1 and all-cause mortality. D: SHR3 and all-cause mortality. All analyses were adjusted for confounding factors including ischemic time, age, sex, BMI, hypertension, diabetes, hyperlipidemia, smoking status, previous CVD, previous AF, previous stroke, CKD, previous HF, cancer, culprit vessel, multi-vessel disease.

Supplementary Material 4
Supplement Figure 2. K-M analyses for all-cause mortality and MACCE.
A: SHR1 and all-cause mortality. B: SHR3 and all-cause mortality. C: SHR1 and MACCE. D: SHR3 and MACCE. MACCE: Major adverse cardiac and cerebrovascular events.

Supplementary Material 5
Supplement Figure 3. K-M analyses for all-cause mortality and MACCE.
A: ABG and all-cause mortality. B: FBS and all-cause mortality. C: ABG and MACCE. D: FBS and MACCE. MACCE: Major adverse cardiac and cerebrovascular events.

Supplementary Material 6
Supplement Figure 4. Subgroup analyses of stress hyperglycemia in normal weight and overweight population.
A: In-hospital death. B: All-cause mortality. All analyses were adjusted for confounding factors including ischemic time, age, sex, DM, hypertension, hyperlipidemia, smoking status, previous CVD, previous AF, previous stroke, CKD, previous HF, cancer, culprit vessel, multi-vessel disease.

Supplementary Material 7
Supplement Figure 5. Subgroup analyses of stress hyperglycemia in hyperlipidemic and non-hyperlipidemic population.
A: In-hospital death. B: All-cause mortality. All analyses were adjusted for confounding factors including ischemic time, age, sex, BMI, DM, hypertension, smoking status, previous CVD, previous AF, previous stroke, CKD, previous HF, cancer, culprit vessel, multi-vessel disease.

Supplementary Material 8
Supplement Figure 6. Subgroup analyses of stress hyperglycemia in different age groups.
A: In-hospital death. B: All-cause mortality. All analyses were adjusted for confounding factors including ischemic time, sex, BMI, DM, hypertension, hyperlipidemia, smoking status, previous CVD, previous AF, previous stroke, CKD, previous HF, cancer, culprit vessel, multi-vessel disease.

## Data Availability

The data analyzed in this study can be obtained from the corresponding author with a reasonable request.

## Abbreviations

CAD: Coronary artery disease
STEMI: ST-elevation myocardial infarction
PCI: Percutaneous coronary intervention
ACS: Acute coronary syndrome
ABG: Admission blood glucose
FBS: Fasting blood sugar
SHR: Stress hyperglycemia ratio
MACCE: Major adverse cardiac and cerebrovascular events
CKD: Chronic kidney disease
DM: Diabetes mellitus
AF: Atrial fibrillation
HF: Heart failure
