# Supplementary material for: Association of stress hyperglycemia with clinical outcomes in patients with ST-elevation myocardial infarction undergoing percutaneous coronary intervention: a cohort study
Source: Cardiovasc Diabetol. 2023 Apr 12;22:85. doi: 10.1186/s12933-023-01812-9 (PMC10100063; doi:10.1186/s12933-023-01812-9)
Supplement: Supplementary file 1 — Supplementary Material 1 [file 12933_2023_1812_MOESM1_ESM.docx]

Supplement Table 1. Multivariable Cox regression and Logistic regression analyses for different end points

|  | In-hospital death | | All-cause mortality | | Unplanned revascularization | | MACCE | |
| --- | --- | --- | --- | --- | --- | --- | --- | --- |
|  | OR (95% CI) | *p* | HR (95% CI) | *p* | HR (95% CI) | *p* | HR (95% CI) | *p* |
| Glucose |  |  |  |  |  |  |  |  |
| Q1 | Reference |  | Reference |  | Reference |  | Reference |  |
| Q2 | 0.95 (0.23, 3.97) | 0.945 | 1.88 (1.02, 3.46) | 0.042 | 0.92 (0.60, 1.42) | 0.716 | 1.14 (0.81, 1.59) | 0.450 |
| Q3 | 3.75 (1.16, 12.17) | 0.028 | 2.06 (1.13, 3.77) | 0.019 | 1.10 (0.72, 1.68) | 0.650 | 1.29 (0.92, 1.81) | 0.136 |
| Q4 | 23.33 (7.34, 74.15) | <0.001 | 5.18 (2.83, 9.50) | <0.001 | 1.05 (0.64, 1.73) | 0.850 | 2.05 (1.43, 2.95) | <0.001 |
| p for trend |  | <0.001 |  | <0.001 |  | 0.711 |  | <0.001 |
| FBS |  |  |  |  |  |  |  |  |
| Q1 | Reference |  | Reference |  | Reference |  | Reference |  |
| Q2 | 0.14 (0.02, 1.13) | 0.065 | 0.96 (0.51, 1.82) | 0.902 | 1.09 (0.70, 1.71) | 0.699 | 0.88 (0.61, 1.25) | 0.459 |
| Q3 | 1.25 (0.44, 3.59) | 0.679 | 1.37 (0.77, 2.46) | 0.287 | 1.63 (1.06, 2.52) | 0.028 | 1.32 (0.95, 1.85) | 0.101 |
| Q4 | 10.10 (3.89, 26.25) | <0.001 | 4.34 (2.45, 7.71) | <0.001 | 1.22 (0.72, 2.08) | 0.457 | 1.94 (1.34, 2.81) | <0.001 |
| p for trend |  | <0.001 |  | <0.001 |  | 0.146 |  | <0.001 |
| SHR1 (per SD) |  |  |  |  |  |  |  |  |
| Q1 | Reference |  | Reference |  | Reference |  | Reference |  |
| Q2 | 8.13 (0.88,75.10) | 0.065 | 2.22 (1.19, 4.13) | 0.012 | 1.00 (0.66, 1.51) | 0.999 | 1.18 (0.84, 1.65) | 0.350 |
| Q3 | 9.69 (1.10, 85.35) | 0.041 | 1.82 (0.97, 3.40) | 0.060 | 0.82 (0.53, 1.29) | 0.394 | 1.05 (0.74, 1.48) | 0.796 |
| Q4 | 20.77 (2.53, 170.73) | 0.005 | 2.05 (1.13, 3.70) | 0.018 | 1.04 (0.69, 1.58) | 0.848 | 1.20 (0.86, 1.66) | 0.288 |
| p for trend |  | <0.001 |  | 0.040 |  | 0.932 |  | 0.410 |
| SHR2 (per SD) |  |  |  |  |  |  |  |  |
| Q1 | Reference |  | Reference |  | Reference |  | Reference |  |
| Q2 | 1.01 (0.13, 7.74) | 0.992 | 1.02 (0.52, 1.97) | 0.961 | 1.30 (0.85, 1.99) | 0.234 | 1.14 (0.80, 1.61) | 0.475 |
| Q3 | 2.14 (0.37, 12.44) | 0.397 | 1.41 (0.75, 2.64) | 0.282 | 1.13 (0.73, 1.75) | 0.585 | 1.13 (0.80, 1.60) | 0.479 |
| Q4 | 9.95 (2.16, 45.74) | 0.003 | 2.39 (1.37, 4.15) | 0.002 | 1.32 (0.85, 2.07) | 0.220 | 1.52 (1.09, 2.13) | 0.014 |
| p for trend |  | <0.001 |  | <0.001 |  | 0.341 |  | 0.019 |
| SHR3 (per SD) |  |  |  |  |  |  |  |  |
| Q1 | Reference |  | Reference |  | Reference |  | Reference |  |
| Q2 | 3.40 (0.36, 31.86) | 0.285 | 0.98 (0.50, 1.90) | 0.948 | 1.20 (0.77, 1.87) | 0.413 | 1.00 (0.70, 1.44) | 0.990 |
| Q3 | 1.64 (0.14, 19.12) | 0.692 | 1.09 (0.57, 2.10) | 0.799 | 1.24 (0.80, 1.91) | 0.339 | 1.07 (0.76, 1.52) | 0.702 |
| Q4 | 19.65 (2.46, 156.66) | 0.005 | 2.55 (1.42, 4.58) | 0.002 | 1.43 (0.90, 2.26) | 0.131 | 1.63 (1.16, 2.31) | 0.005 |
| p for trend |  | <0.001 |  | <0.001 |  | 0.143 |  | 0.006 |

Adjust for ischemia time, age, sex, BMI, hypertension, diabetes, hyperlipidemia, smoking status, previous CVD, previous AF, previous stroke, CKD, previous HF, cancer, culprit vessel, multi-vessel disease.

Abbreviations: ABG: Admission blood glucose; FBS: Fasting blood sugar; SHR: Stress hyperglycemia ratio; MACCE: Major adverse cardiac and cerebrovascular events.
