# Supplementary material for: Association of stress hyperglycemia with clinical outcomes in patients with ST-elevation myocardial infarction undergoing percutaneous coronary intervention: a cohort study
Source: Cardiovasc Diabetol. 2023 Apr 12;22:85. doi: 10.1186/s12933-023-01812-9 (PMC10100063; doi:10.1186/s12933-023-01812-9)
Supplement: Supplementary file 2 — Supplementary Material 2 [file 12933_2023_1812_MOESM2_ESM.docx]

Supplement Table 2. Multivariable Cox regression and Logistic regression analyses for different end points

|  | In-hospital death | | All-cause mortality | | Unplanned revascularization | | MACCE | |
| --- | --- | --- | --- | --- | --- | --- | --- | --- |
|  | OR (95% CI) | *p* | HR (95% CI) | *p* | HR (95% CI) | *p* | HR (95% CI) | *p* |
| ABG |  |  |  |  |  |  |  |  |
| Non-hyperglycemia | Reference |  | Reference |  | Reference |  | Reference |  |
| Hyperglycemia | 6.23 (2.79, 13.89) | <0.001 | 1.90 (1.28, 2.82) | 0.001 | 1.04 (0.74, 1.46) | 0.809 | 1.26 (0.99, 1.62) | 0.065 |
| FBS |  |  |  |  |  |  |  |  |
| Non-hyperglycemia | Reference |  | Reference |  | Reference |  | Reference |  |
| Hyperglycemia | 12.27 (6.01, 25.03) | <0.001 | 3.81 (2.50, 5.79) | <0.001 | 0.99 (0.64, 1.51) | 0.949 | 1.82 (1.35, 2.44) | <0.001 |

Adjust for ischemia time, age, sex, BMI, hypertension, diabetes, hyperlipidemia, smoking status, previous CVD, previous AF, previous stroke, CKD, previous HF, cancer, culprit vessel, multi-vessel disease.

Abbreviations: ABG: Admission blood glucose; FBS: Fasting blood sugar; MACCE: Major adverse cardiac and cerebrovascular events.
