# Supplementary figures and images for: Association of stress hyperglycemia with clinical outcomes in patients with ST-elevation myocardial infarction undergoing percutaneous coronary intervention: a cohort study
Source: Cardiovasc Diabetol. 2023 Apr 12;22:85. doi: 10.1186/s12933-023-01812-9 (PMC10100063; doi:10.1186/s12933-023-01812-9)

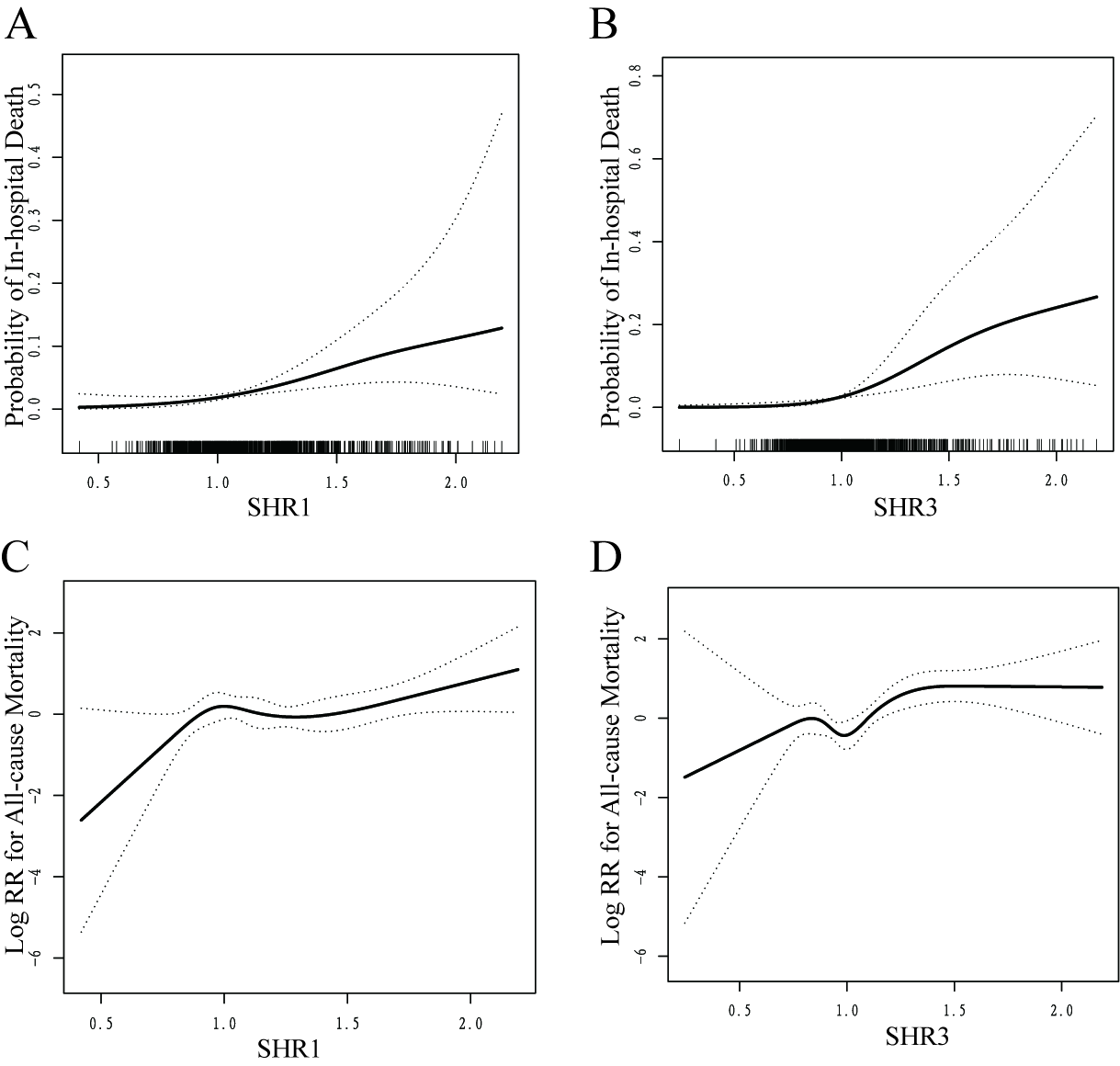

Supplement: Supplementary file 3 — Supplementary Material 3 Supplement Figure 1. Association of stress hyperglycemia and adverse outcomes. A: SHR1 and in-hospital death. B: SHR3 and in-hospital death. C: SHR1 and all-cause mortality. D: SHR3 and all-cause mortality. All analyses were adjusted for confounding factors including ischemic time, age, sex, BMI, hypertension, diabetes, hyperlipidemia, smoking status, previous CVD, previous AF, previous stroke, CKD, previous HF, cancer, culprit vessel, multi-vessel disease. [file 12933_2023_1812_MOESM3_ESM.tif]

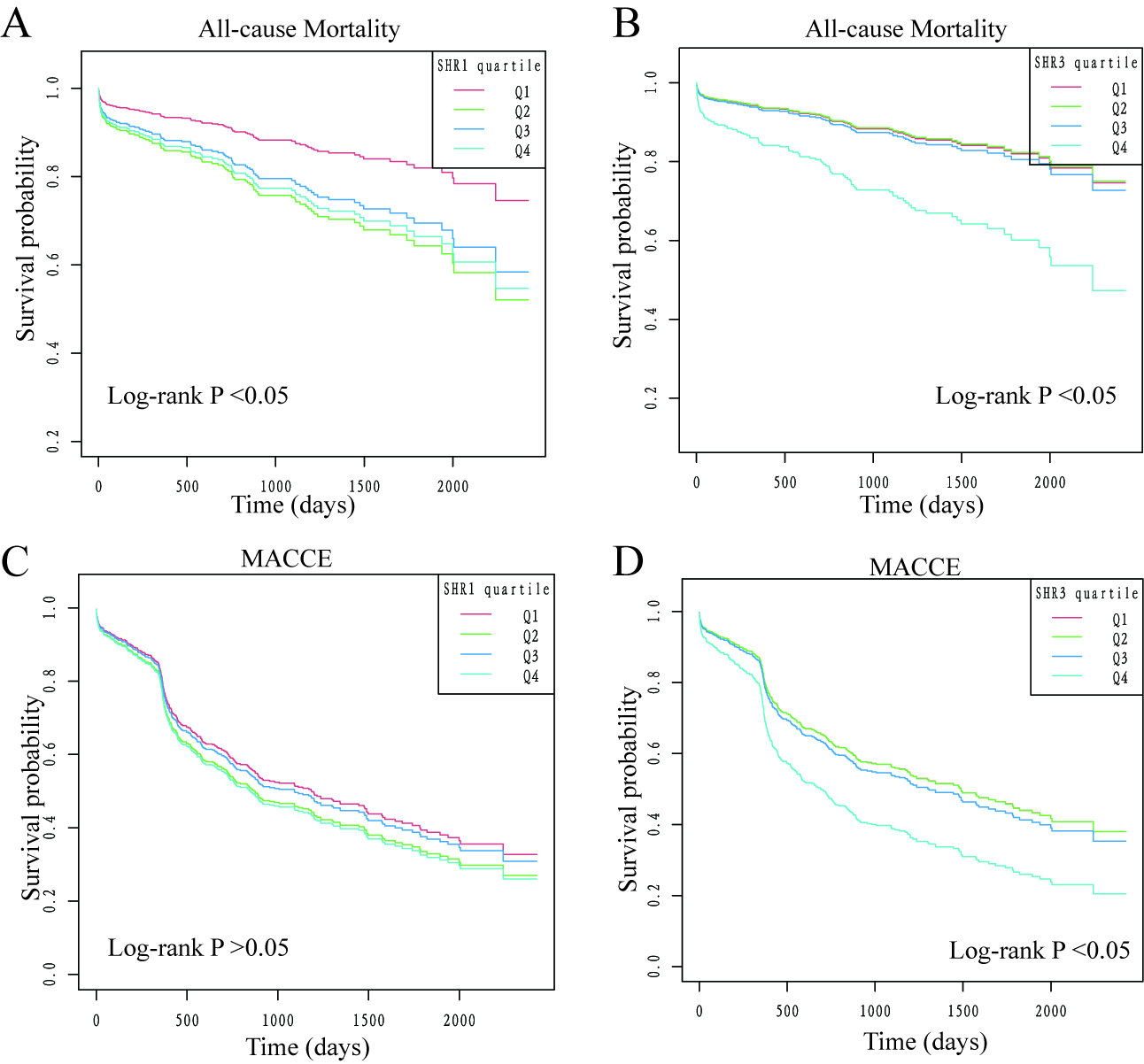

Supplement: Supplementary file 4 — Supplementary Material 4 Supplement Figure 2. K-M analyses for all-cause mortality and MACCE. A: SHR1 and all-cause mortality. B: SHR3 and all-cause mortality. C: SHR1 and MACCE. D: SHR3 and MACCE. MACCE: Major adverse cardiac and cerebrovascular events. [file 12933_2023_1812_MOESM4_ESM.tif]

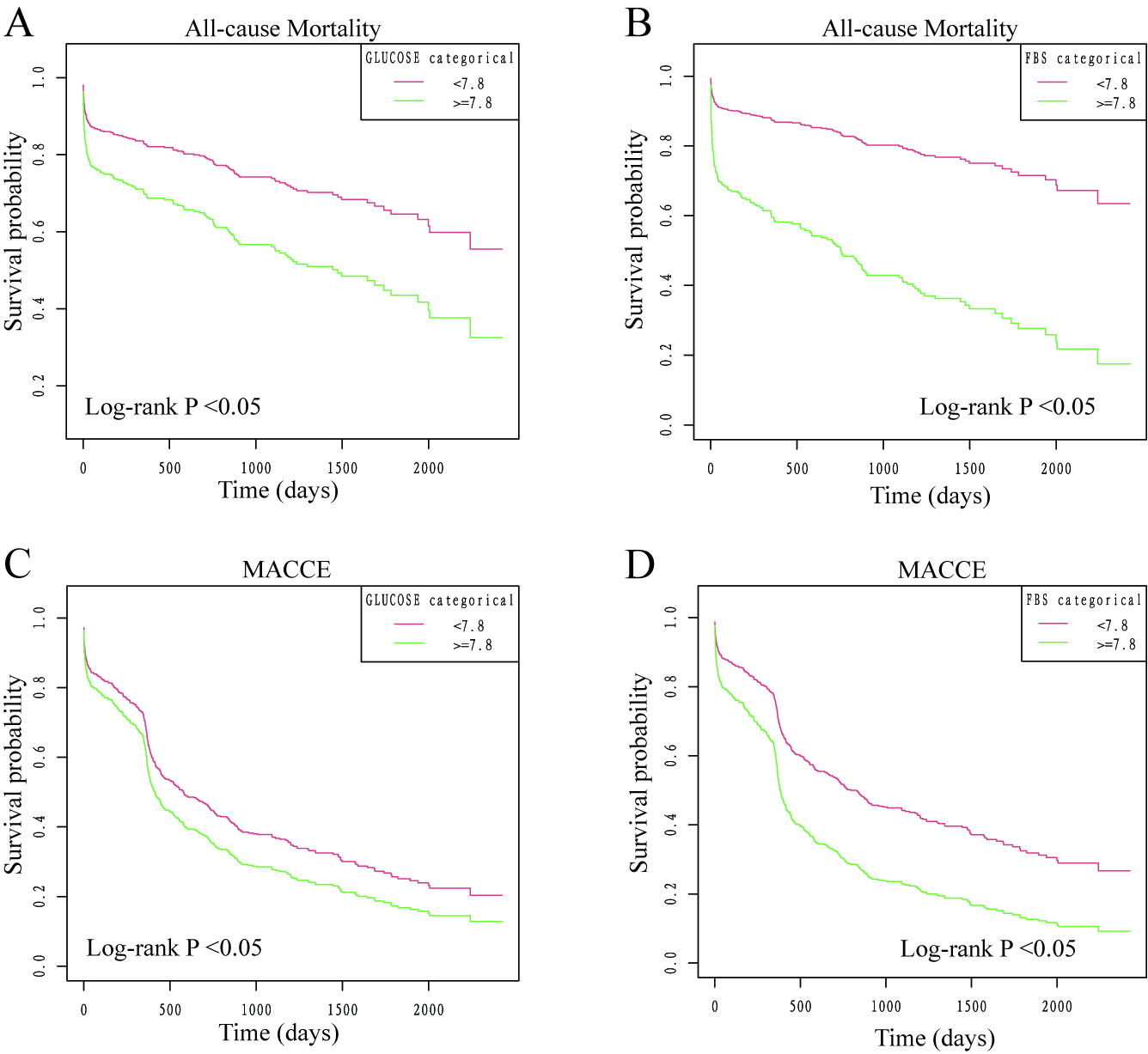

Supplement: Supplementary file 5 — Supplementary Material 5 Supplement Figure 3. K-M analyses for all-cause mortality and MACCE. A: ABG and all-cause mortality. B: FBS and all-cause mortality. C: ABG and MACCE. D: FBS and MACCE. MACCE: Major adverse cardiac and cerebrovascular events. [file 12933_2023_1812_MOESM5_ESM.tif]

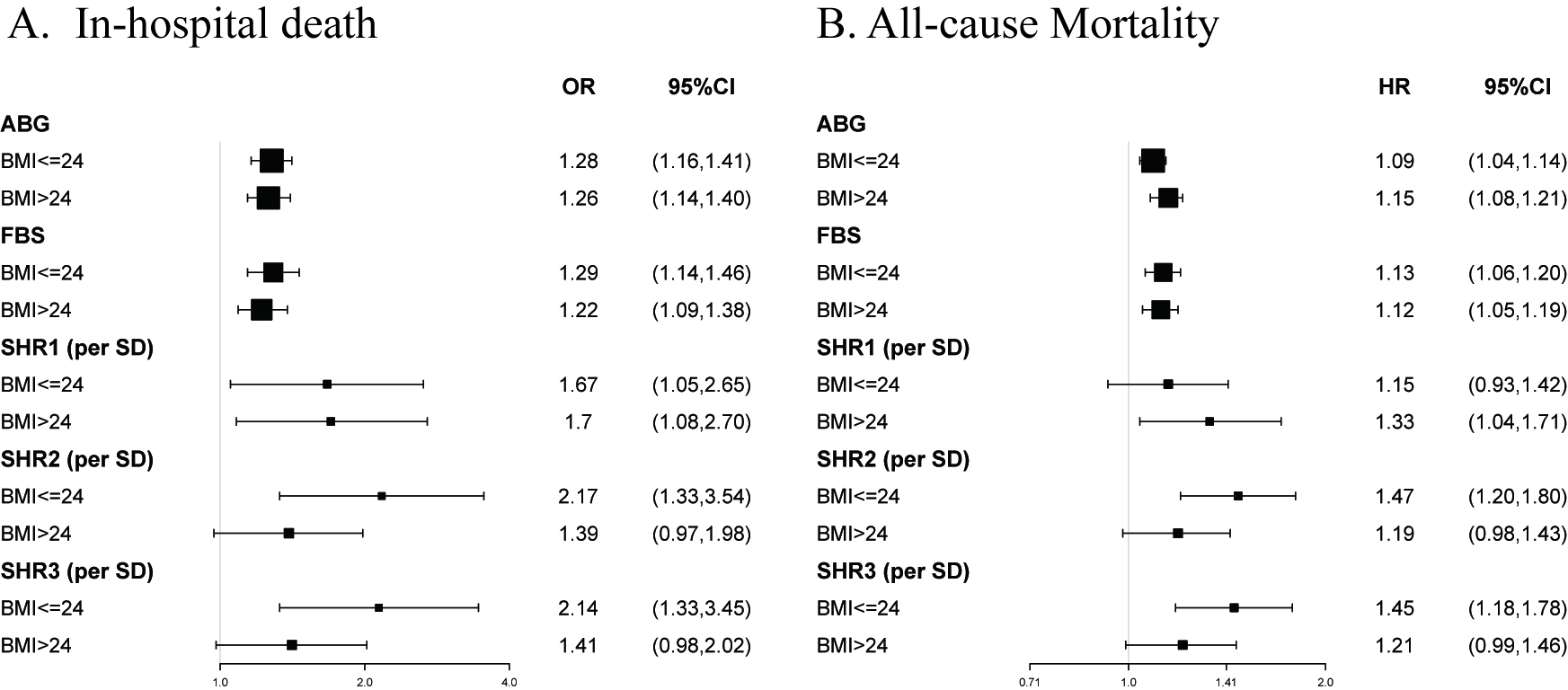

Supplement: Supplementary file 6 — Supplementary Material 6 Supplement Figure 4. Subgroup analyses of stress hyperglycemia in normal weight and overweight population. A: In-hospital death. B: All-cause mortality. All analyses were adjusted for confounding factors including ischemic time, age, sex, DM, hypertension, hyperlipidemia, smoking status, previous CVD, previous AF, previous stroke, CKD, previous HF, cancer, culprit vessel, multi-vessel disease. [file 12933_2023_1812_MOESM6_ESM.tif]

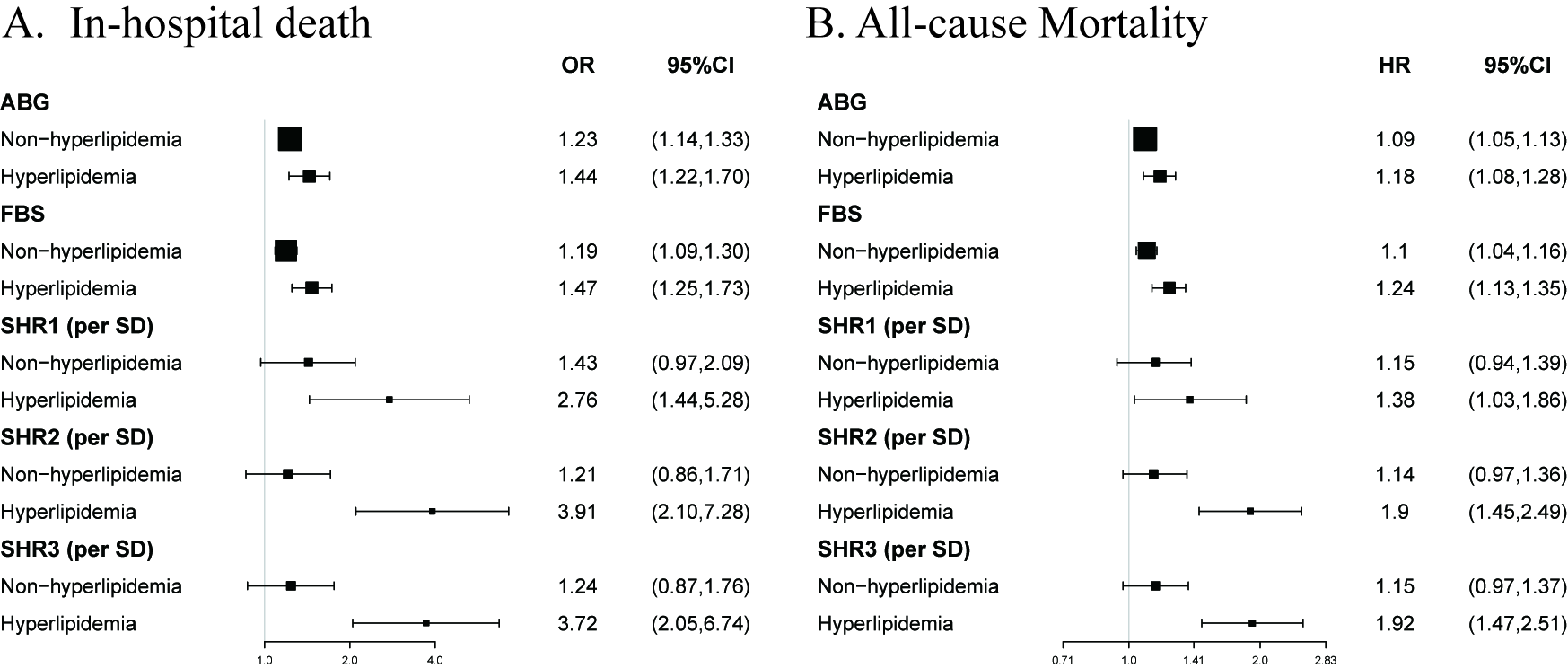

Supplement: Supplementary file 7 — Supplementary Material 7 Supplement Figure 5. Subgroup analyses of stress hyperglycemia in hyperlipidemic and non-hyperlipidemic population. A: In-hospital death. B: All-cause mortality. All analyses were adjusted for confounding factors including ischemic time, age, sex, BMI, DM, hypertension, smoking status, previous CVD, previous AF, previous stroke, CKD, previous HF, cancer, culprit vessel, multi-vessel disease. [file 12933_2023_1812_MOESM7_ESM.tif]

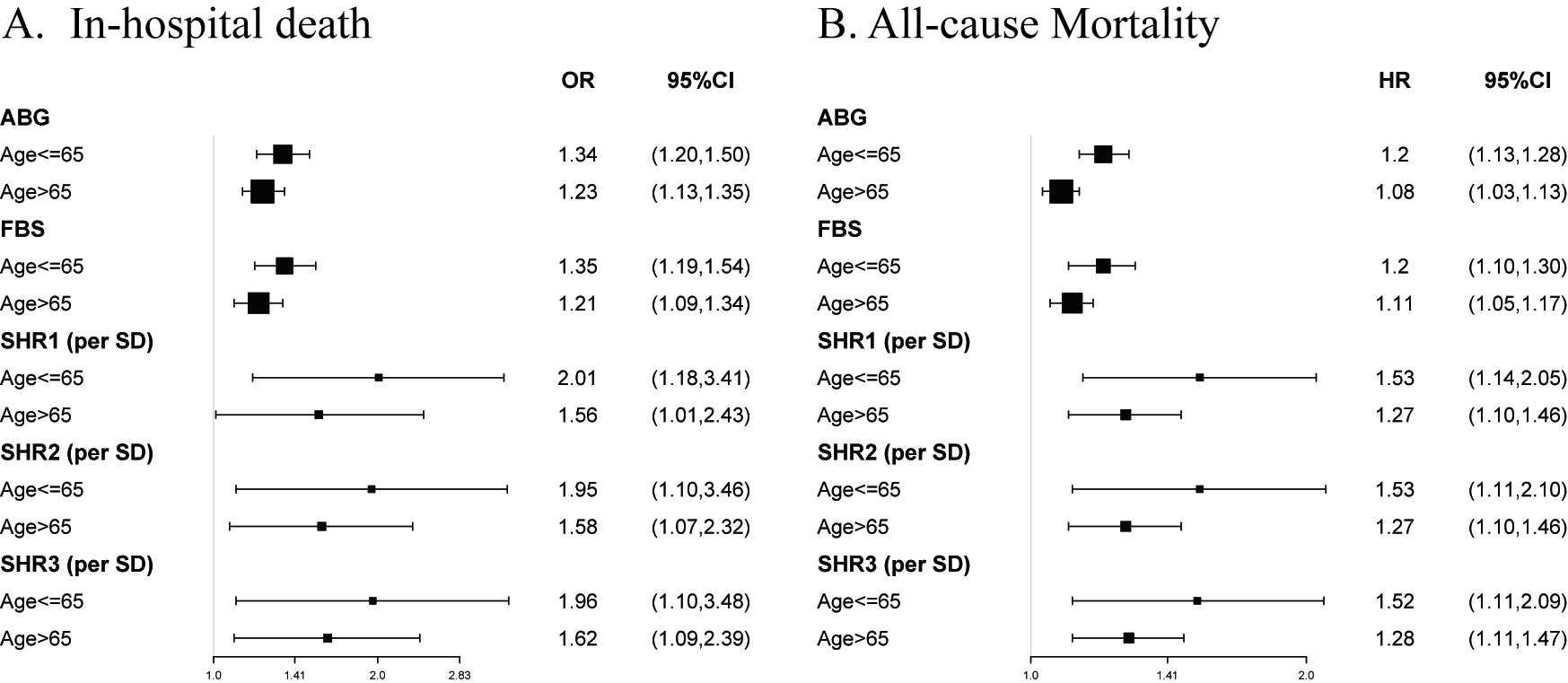

Supplement: Supplementary file 8 — Supplementary Material 8 Supplement Figure 6. Subgroup analyses of stress hyperglycemia in different age groups. A: In-hospital death. B: All-cause mortality. All analyses were adjusted for confounding factors including ischemic time, sex, BMI, DM, hypertension, hyperlipidemia, smoking status, previous CVD, previous AF, previous stroke, CKD, previous HF, cancer, culprit vessel, multi-vessel disease. [file 12933_2023_1812_MOESM8_ESM.tif]
